# Reply to: Revisiting the identification of *Syllipsimopodi bideni* and timing of the decabrachian-octobrachian divergence

**DOI:** 10.1038/s41467-023-42843-w

**Published:** 2023-12-12

**Authors:** Christopher D. Whalen, Neil H. Landman

**Affiliations:** 1 American Museum of Natural History, 200 Central Park West, New York, NY 10024 USA; 2 Richard Gilder Graduate School, New York, NY USA; 3 Yale University, 210 Whitney Ave., New Haven, CT 06511 USA

**Keywords:** Taxonomy, Palaeontology, Phylogenetics

**replying to** C. Klug et al. *Nature Communications* 10.1038/s41467-023-42842-x (2023)

Bayesian tip-dating recovered the Carboniferous (Serpukhovian) coleoid *Syllipsimopodi bideni* Whalen and Landman 2022^1^ (Fig. 1) as the earliest diverging vampyropod^1^. The analysis thus suggests that vampyropods (=total group), octobrachians (=superorder), and octopodiforms (=crown group) diverged from decabrachians (=superorder) and decapodiforms (=crown group) in the Mississippian^1^; this agrees with several molecular divergence time estimates^2–5^, but suggests an older split than some others^6,7^. Considering the divergence-time implications, Klug et al.^8^ suggest *Syllipsimopodi* is a junior synonym of the stem neocoleoid *Gordoniconus beargulchensis* Mapes et al.^9^ (Fig. 2). However, divergence-times calibrated using the then-oldest-known fossil do not preclude discovery of an older fossil. Node-dated analyses constrain divergence times using a set of user-defined calibrations; e.g., López-Córdova et al.^7^ recovered a Middle Triassic octobrachian-decabrachian divergence after assigning that node to the Middle Triassic *Germanoteuthis*. Whalen and Landman^1^ conducted the first tip-dated coleoid analysis. Tip-dating does not require node calibrations; instead, the node dates and interrelationships are simultaneously inferred based on the dates/characters of all terminal taxa included in the analysis. For this reason, poorly-known taxa, such as *Pohlsepia*, were explicitly excluded from consideration and thus had no impact on Whalen and Landman’s^1^ analysis, contra Klug et al.^8^. Rather than conducting a new phylogenetic analysis that supports a different position for *Syllipsimopodi* in the coleoid tree, Klug et al.^8^ instead challenge the validity of *Syllipsimopodi*. However, *Gordoniconus* and *Syllipsimopodi* have fundamental morphological differences suggesting they should be maintained as distinct taxa.

**Fig. 1:**
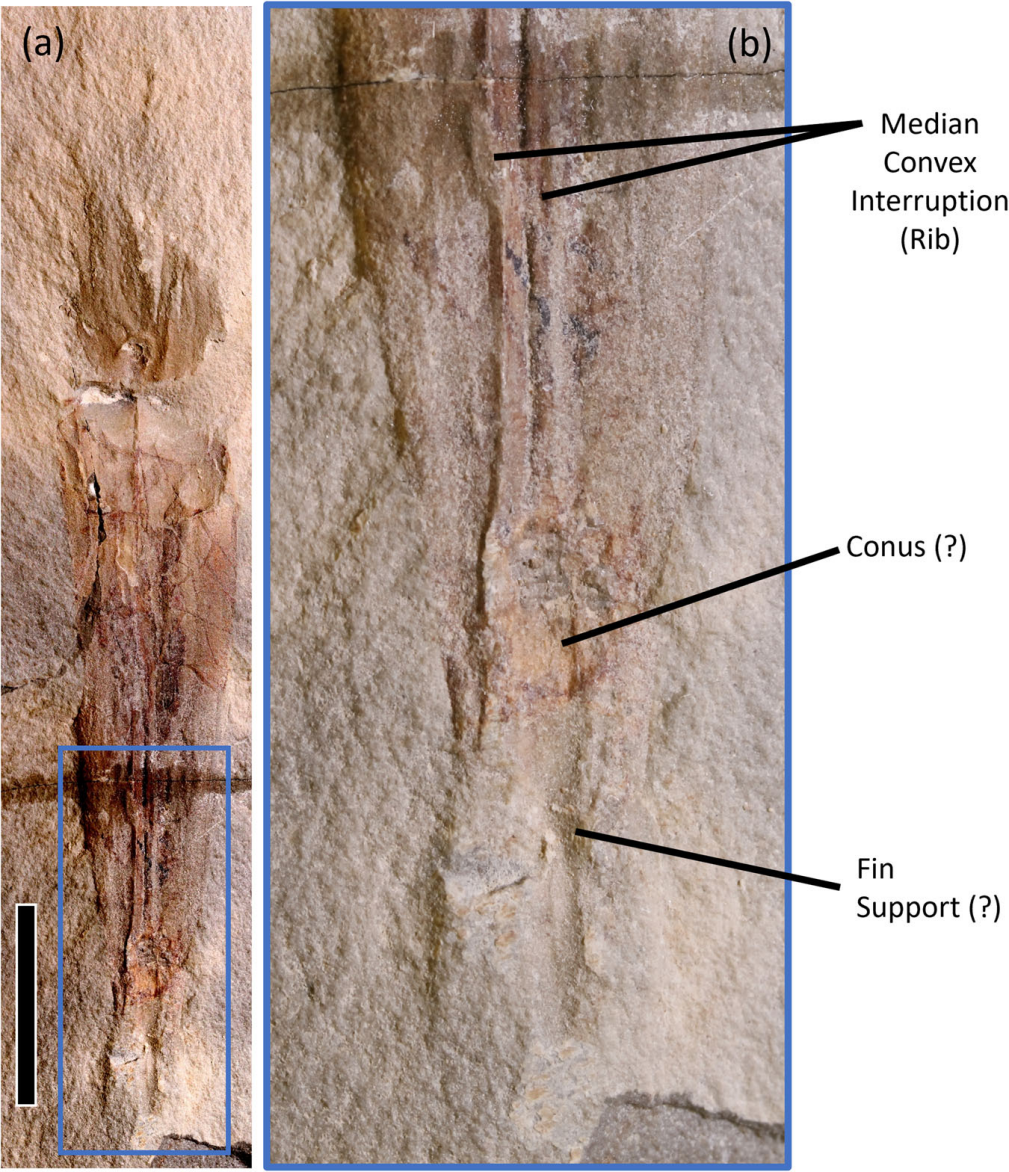
*Syllipsimopodi bideni* holotype ROMIP 64897. **a** Full specimen; scale = 1 cm. **b** Boxed region of Fig. 1a, showing posterior/apex. Note the pronounced median convex interruption (posteriorly bipartite rib), possible conus, and possible fin support; also note the absence of septa and a primordial rostrum (compare to Fig. 2c). Color differences between images caused by lighting angle.

**Fig. 2:**
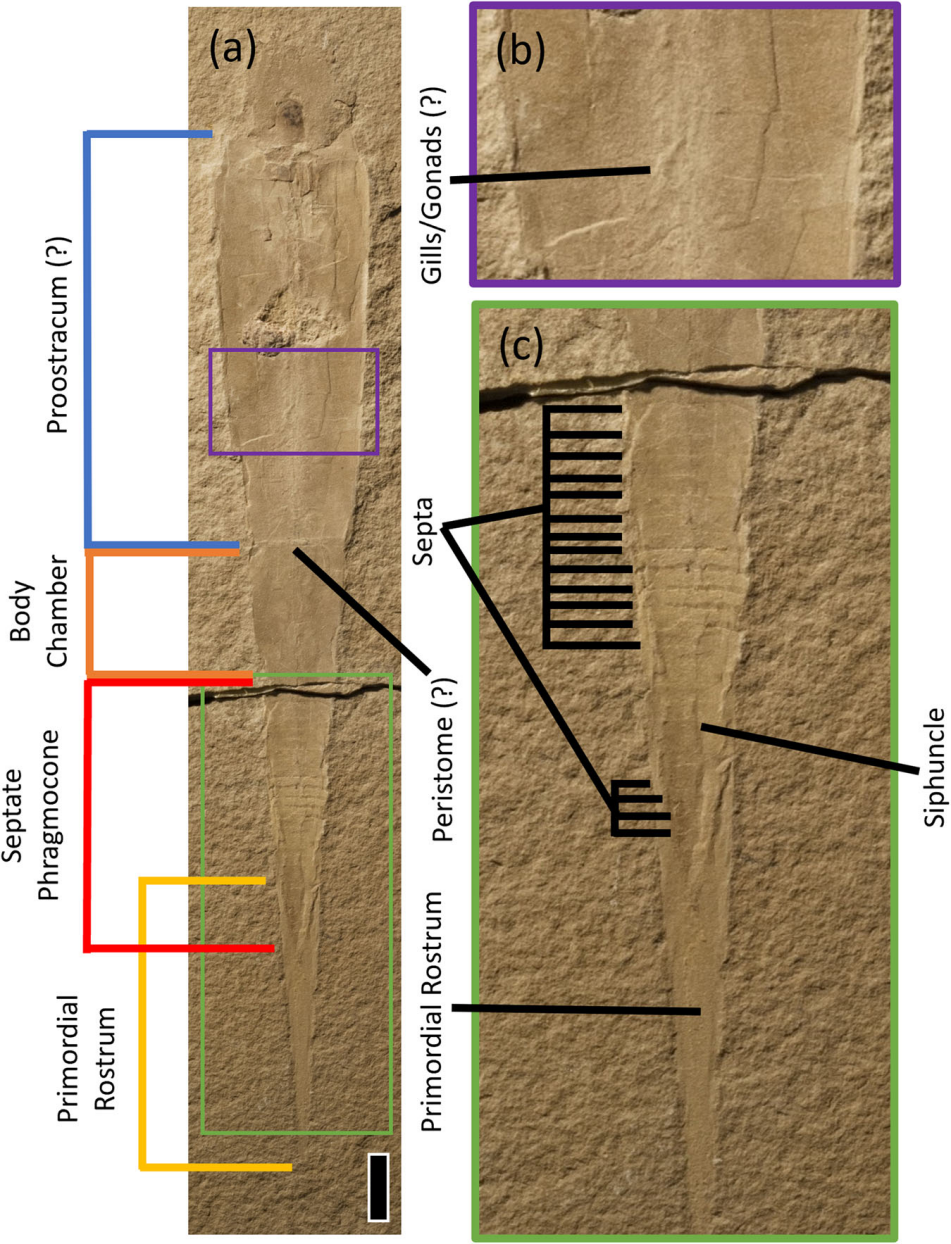
*Gordoniconus beargulchensis* holotype AMNH 43264, part. **a** Complete specimen, showing suggested proostracum (blue), body chamber (orange), septate phragmocone (red) and primordial rostrum (yellow); scale = 1 cm. **b** Purple boxed region of Fig. 1a, showing structure interpreted as gills/gonads. **c** Green boxed posterior region of Fig. 1a, showing phragmocone and primordial rostrum; note the thin siphuncle and numerous complete septa, not all are marked. Image credit: S. Thurston.

The *Syllipsimopodi* (ROMIP 64897) and *Gordoniconus* (AMNH 43264/50267) holotypes are both from the Bear Gulch lagerstätte; therefore, both fossils are likely subject to similar taphonomic biases and differences between genera are unlikely to be preservational artifacts. Klug et al.^8^ accept several fragmentary fossils as specimens of *Gordoniconus* (i.e., CM 52637, 52640, 52658)^10^; this suggests a broader array of preservational/anatomical differences than we consider reasonable. We do not think there is sufficient evidence to assign these specimens to *Gordoniconus* at this time. Future inquiry may demonstrate that some of these supposed *Gordoniconus* specimens are actually new genera, or perhaps specimens of *Syllipsimopodi*. Our analysis is based on direct examination of the non-fragmentary and extremely well-preserved type specimens.

*Gordoniconus* possesses a septate phragmocone and primordial rostrum (= ‘rostrum’ in^8,10^); these are not observed in *Syllipsimopodi*. It is unclear to us what is referenced on ROMIP 64897 (Fig. 1) to support the illustrated septa of Klug et al. Fig. 1b^8^. As noted by Whalen and Landman^1^, it is unlikely for septa to have dissolved without leaving any trace when they are clearly observable in co-occurring *Gordoniconus*. Thus, if septa were present, they must have been unmineralized or poorly mineralized in vivo – a clear difference from *Gordoniconus*. Klug et al.^8^ reinterpret the suggested fin support of *Syllipsimopodi* as a siphuncle, which is not implausible. However, this ~ 2.4mm-wide structure is much larger than the ~1mm-wide siphuncle of *Gordoniconus* (Fig. 2b), despite *Gordoniconus* being the larger specimen. As an explanation for the missing primordial rostrum, Klug et al.^8^ suggest ROMIP 64897 is posteriorly damaged; this is possible.

*Syllipsimopodi* uncontestedly^8^ possesses suckers^1^, which have not yet been observed in any *Gordoniconus* specimen. *Syllipsimopodi* clearly possesses a proostracum/gladius (a gladius is simply a proostracum without a mineralized phragmocone), as evidenced by the high-angle growth lines (Supplementary Fig. 3 in ref. ^1^) and median convex interruption or rib (Fig. 1). Neither piece of evidence is compatible with a body chamber or phragmocone. Whalen and Landman^1^ code a simple proostracum as present in *Gordoniconus*, though evidence here is less clear and Klug et al.^8^ advocate its absence. If Klug et al.^8^ are correct, then the presence/absence of a proostracum is a defining difference between *Syllipsimopodi* and *Gordoniconus*. If Whalen and Landman^1^ are correct, then the two genera possess very different proostraca.

A median convex interruption (rib) is either absent (*Gordoniconus*) or present (*Syllipsimopodi*); we are not aware of any cephalopods that are polymorphic for this trait. It alone is sufficient to justify taxonomic separation. The rib is a pronounced topographic structure that cannot be easily lost taphonomically, obscured diagenetically, or misidentified. *Gordoniconus* has a faint and complex median structure interpreted as gills/gonads^10^ (Fig. 2). These are clearly distinct from the raised rib of *Syllipsimopodi* (Fig. 1). Klug et al.^8^ depict the rib as a wavy ‘median crack’ in their line-drawing (Fig. 1b^8^); this is inaccurate. The rib is not irregular or wavy and it has no jagged edges or broken surfaces; it is a straight, smooth, bipartite raised structure (Fig. 1).

Whalen and Landman^1^ provided a detailed camera lucida drawing of *Syllipsimopodi* (Supplementary Fig. 2 in ref. ^1^). This was not referenced by Klug et al.^8^, who use a simplified line-drawing based on Whalen and Landman Supplementary Fig. 6 in ref. ^1^; this line-drawing does not faithfully represent all structures (e.g., rib) or the fossil outline. Klug et al.^8^ overlay line drawings of *Syllipsimopodi* and *Gordoniconus* to highlight perceived similarities. Unsurprisingly, the edges roughly coincide when *Gordoniconus* is shrunken and the apices unaligned. Similar attempts could include numerous distinct triangles/cones; by this rationale many well-established early coleoids would be synonymized (e.g., *Donovaniconus, Saundersites, Mutveiconites, Flowericonus*). Excluding appendages, *Syllipsimopodi* is ~8.3 cm-long (including possible fin) and ~1.9 cm-wide; *Gordoniconus* is ~15.3 cm-long and ~ 2.1 cm-wide (widths measured at shell anterior). Length-to-width ratios are ~4.4 (*Syllipsimopodi*) and ~ 7.3 (*Gordoniconus*). Excluding the two seemingly-elongate appendages of *Syllipsimopodi* from both arm and body lengths, the arms of *Syllipsimopodi* are ~20.3% of body-length; the arms of *Gordoniconus* are ~ 10.0% of total-body-length. The angle between the shell’s anterior margin and anterolateral margin is ~ 75° for *Syllipsimopodi* and ~ 95° for *Gordoniconus*. The length of *Syllipsimopodi* could be affected by a possible missing posterior, but the shell anterior is completely preserved (given the appendages), so width and angle measurements should be uncontroversial. Therefore, contra Klug et al.^8^, measurements/metrics support the validity of *Syllipsimopodi* as a separate taxon. To explain absolute size differences, Klug et al.^8^ assert (without explanation) that ROMIP 64897 represents a ‘different’ ontogenetic stage than all other known *Gordoniconus*. Considering the numerous differences between *Syllipsimopodi* and *Gordoniconus*, and the absence of fossils representing intervening stages, we consider this unlikely.

Soft-bodied fossils are regularly subject to competing anatomical interpretations, and the first word on a novel taxon is rarely the last. However, Klug et al.^8^ have not provided new materials or new analyses; their opinion quietly disregards evidential characters demonstrating the distinctiveness of each genus and invokes possible structures for which evidence is lacking. To some extent, this debate is a result of our different ideas for what constitutes a plausible anatomical interpretation for a Carboniferous coleoid. Klug et al.^8^ favor a later octobrachian-decabrachian divergence and the traditional homology framework for the proostracum, while Whalen and Landman^1^ cite their tip-dated analysis as a refinement of the node-dated divergence times and explicitly reject the traditional homology framework in light of new evidence (e.g.,^1,11–13^). Klug et al.^8^ remind us that *Syllipsimopodi*, *Gordoniconus*, and other early coleoids deserve further study to clarify their morphologies and systematic affinities, and additional studies are, indeed, underway.

Based on side-by-side and microscopic examination of the complete and well-preserved holotypes, we maintain that *S. bideni* is not synonymous with *G. beargulchensis*, nor should *S. bideni* be transferred to *Gordoniconus*. The two genera are, in fact, so distinct that they do not form a clade in the coleoid phylogeny^1^, a straightforward prerequisite for synonymy. Phylogenies are hypotheses and future analyses will determine whether *Syllipsimopodi* remains a vampyropod, but it is a valid genus.

## Methods

The holotypes, ROMIP 64897 and AMNH 43264/50267, were observed using a hand lens and under a light microscope at the American Museum of Natural History. The photograph for Fig. 1a was taken using a Canon EOS 60D camera with an EF-S60mm f/2.8 Macro USM lens and a Hoya 52 mm Circular Polarizing Pro 1 digital multi-coated glass filter; Cognisys Stackshot 3X Macro Rail Package and Helicon Focus 6.7.1 Pro were used to z-stack images. Photographs for Fig. 1b and Fig. 2 were taken using a Nikon D300 camera. Composite images were stitched using Adobe Photoshop 2021. All measurements were taken using ImageJ.

## Data Availability

All data generated or analysed during this study (i.e., measurements, observations, photographs) are included in the text and figures of this published article. ROMIP 64897 is reposited at the Royal Ontario Museum (Toronto, ON, CA) and AMNH 43264/50267 is reposited at the American Museum of Natural History (New York, NY, USA).
